# Supplementary material for: Chromothripsis during telomere crisis is independent of NHEJ, and consistent with a replicative origin
Source: Genome Res. 2019 May;29(5):737–49. doi: 10.1101/gr.240705.118 (PMC6499312; doi:10.1101/gr.240705.118)
Supplement: Supplemental Material [file supp_gr.240705.118_Supplemental_file_1.zip › contigs/annotated_contigs/DB113/contig.2.DB113_length_765_mean_cov_10.9803921569.docx]

**DB113_length_765_mean_cov_10.9803921569**

```
AAGACTTTCTTTAGTTTTGGCATTTTTAATGCTTTTTGGCAACAACTAACATACCCCACGAAAAATGGATTAAATAGAGGACTTCAAAT
 >chr12:41875755-41876227 + E=4e-269 p=5e-02
TCATTTTGATCATATCAAAACATTCCACAAATAACTGTTCCTAGCTTGGATCAGTGAATCACTGATGTCTGACCATTGGGTTGCCTTCT

CTTTCTTTTAGACTTTTCCTCATTGTCACAAAGGGGCTGCCACAGCTGTAAGCATCACATCTTCATGCAGCAAAACCTAAGGTGGAAAG

AAAGAGAAGGTGTTTTCCTTGTCATCTCTCTCTGATATCAAAATGAAAAATCCTAGATATGCCTCAGCAGATTCCCACAGAAATACTTT

GGACCAGTGTAGGATTGTATATGCTTGTAGCTATCAAGGAAGTTGGAAAAGAAAAATCTGAAATATTTAGTTTAGGATGAGAACTTGGA

TTCTACAACAAGGAAAAAAAGGAA|GAG|ACATAATTTACATGAGATCTAAAGCACAGCCTATATTCCAGCTCAGCCTTTAAATATTCT
                         >chr12:41876880-41877176 + E=3e-163
TACCCTTTTATATTTTCCCCTCTTTATGAAAGAATAGATAGTAGGTGACCTCCACAACCAAAGTTGATCATTTCTTCTTCATTTTTCAA

TTTCCTTTTTTAAGGTTATCAAAAAGTAGCAATTCATTTTCATTCTGTCTATGAACTTACACAAGATCCTACACAGTAAGCTTACTAAA

AGAGAAGGATATCAAGAAGAGTGAAAAATAATATATCTCTTTTGAAATTTCTTAG
```
